# The effects of beetroot juice supplementation on exercise economy, rating of perceived exertion and running mechanics in elite distance runners: A double-blinded, randomized study

**DOI:** 10.1371/journal.pone.0200517

**Published:** 2018-07-11

**Authors:** Carlos Balsalobre-Fernández, Blanca Romero-Moraleda, Rocío Cupeiro, Ana Belén Peinado, Javier Butragueño, Pedro J. Benito

**Affiliations:** 1 Department of Physical Education, Sport and Human Movement, Universidad Autónoma de Madrid, Madrid, Spain; 2 Laboratory of Exercise Physiology Research Group, Department of Health and Human Performance, School of Physical Activity and Sport Sciences-INEF, Universidad Politecnica de Madrid, Madrid, Spain; 3 Faculty of Health, Camilo José Cela University, Madrid, Spain; University of Alabama at Birmingham, UNITED STATES

## Abstract

**Purpose:**

Nitrate-rich beetroot juice supplementation has been extensively used to increase exercise economy in different populations. However, its use in elite distance runners, and its potential effects on biomechanical aspects of running have not been properly investigated. This study aims to analyze the potential effects of 15 days of beetroot juice supplementation on physiological, psychological and biomechanical variables in elite runners.

**Methods:**

Twelve elite middle and long-distance runners (age = 26.3 ± 5.1yrs, VO_2_Max = 71.8±5.2 ml*kg^-1^*min^-1^) completed an incremental running test to exhaustion on a treadmill before and after a 15-days supplementation period, in which half of the group (EG) consumed a daily nitrate-rich beetroot juice and the other group (PG) consumed a placebo drink. Time to exhaustion (TEx), running economy, vastus lateralis oxygen saturation (SmO_2_), leg stiffness and rate of perceived exertion (RPE) were measured at 15, 17.1 and 20 km/h during the incremental test.

**Results:**

Likely to very likely improvements in EG were observed for the RPE (Standardized mean difference (SMD) = -2.17, 90%CI = -3.23, -1.1), SmO_2_ (SMD = 0.72, 90%CI = 0.03, 1.41) and TEx (SMD = 1.18, 90%CI = -0.14, 2.5) in comparison with PG. No other physiological or biomechanical variable showed substantial improvements after the supplementation period.

**Conclusions:**

Fifteen days of nitrate-rich beetroot juice supplementation produced substantial improvements in the time to exhaustion in elite runners; however, it didn’t produce meaningful improvements in running economy, VO_2_Max or mechanical parameters.

## Introduction

Performance at the highest level of competition in endurance events depends on several physiological, psychological and biomechanical parameters [1–5]. Even marginal gains in some variables might represent a competitive edge to win the race or break records; for instance, wearing energy-recovering shoes have shown to increase running economy by about 4%, which might have helped in the recent first sub-2h attempt on the marathon [6]. Maximal oxygen consumption is one of the most relevant physiological variables in distance running performance [7,8]; however, in elite athletes other variables seem to influence competitive success to a greater extent [4,9]. Among them, running economy (i.e., the oxygen consumption at a certain pace) has been proposed as one of the most important variables in distance running performance. Theoretically, better running economy means either being able to run at the same pace with a lower exertion or, for the same energy consumption, being able to run faster [9,10]. Therefore, researchers and trainers are focused now on finding strategies to increase economy in different kinds of endurance athletes such as runners, cyclists or swimmers. In this sense, different studies have shown that heavy-loaded resistance training produces a meaningful increase in running economy of athletes at different levels, including elite participants [11,12]. Besides training interventions, different nutritional strategies have also been used to increase distance running performance, from high-carbohydrate to ketogenic diets [13–15]. However, during the last decade one particular supplement has been investigated as a potential beneficial aid for endurance events: beetroot juice [16,17]. Beetroot juice is a high source of nitrate (NO_3_^-^) that has been proposed to increase NO (nitric oxide) availability, a potent signaling molecule that can increase vasodilatation, mitochondrial respiration or glucose uptake, among other factors related to exercise performance [17]. Several studies have shown that beetroot juice supplementation for about a week does have an ergogenic effect in moderate trained runners, mainly via an increase in running economy [16,18,19]. However, there is a lack of studies analyzing its potential benefits in elite subjects, who might need a higher dose or exposure than recreational athletes to obtain the desired increases in performance. A recent study [20] observed that 8 days of beetroot supplementation didn’t increase performance variables in elite 1500m runners; however, whether larger interventions might have benefits in elite populations is unknown. Moreover, to the best of our knowledge, no studies have analyzed the potential effects of beetroot supplementation in mechanical aspects of running such as leg stiffness, a variable that has been linked to running economy in different studies [21–23]. For this, the purpose of this study is to analyze the physiological, psychological and biomechanical effects of beetroot juice supplementation in elite distance runners.

## Materials & methods

### Participants

Twelve elite middle and long-distance runners, including European medalists and Olympians were recruited from the High Performance Center of Madrid, Spain to join this investigation. Inclusion criteria were as follows: male competitors in national and international events, with personal bests in the urban 10k below 32 min, and below 3min50s. in the 1500m. See details in Table 1. Prior to commencement, all participants provided written informed consent. Participants were instructed to not consume beetroot of any form 2 months before the start of the intervention and to maintain their normal diet throughout the testing period, to follow the same diet for 24 hours prior to each trial, to avoid food and drink in the hour before each trial, and to refrain from strenuous exercise for 24 hours before each trial. None of the participants followed a plant-based (vegetarian or vegan) diet, and they never consumed nitrate-rich beetroot juice in the past. The study protocol complied with the Declaration of Helsinki for Human Experimentation and was approved by the Institutional Review Board of the Universidad Camilo José Cela.

**Table 1:** Characteristics of the participants. Personal bests are expressed as range.

	Age (yrs.)	Height (cm)	Weight (kg)	VO_2_Max (ml*kg^-1^*min^-1^)	PB in urban 10 km (min:s)	PB in outdoor 1500m (min:s)
Experimental group	27.3±7.8	1.79±0.07	69.2±8.6	69.1±5.3	29:22–31:00	3:32–3:45
Control group	24.2±2.9	1.78±0.04	65.2±2.6	72.3±6.8	28:28–30:54	3:43–3:45

### Procedures

#### Beetroot juice supplementation protocol

**Fig 1:**
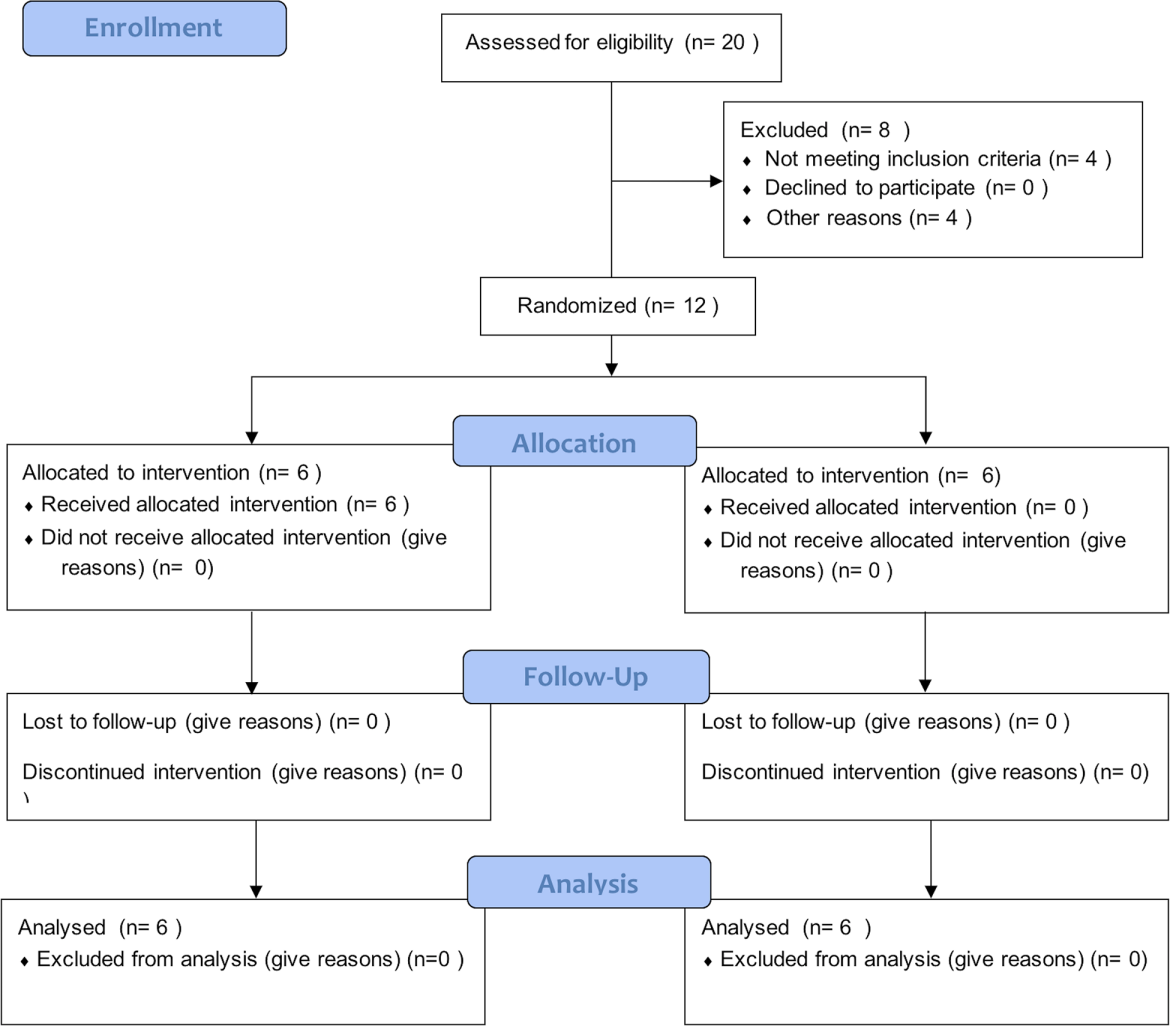
CONSORT flowchart.

Participants were randomly assigned to either a placebo (PG) or an experimental (EG) group in a double-blinded manner so neither the athletes nor the main researchers could possibly identify who were consuming a nitrate-rich beetroot juice supplementation. See Fig 1.

Participants in EG consumed a high-nitrate beetroot juice (6.5-mmol NO3^-^/70-mL, Beet It Sport; James White Drinks, Ipswich, United Kingdom) for 15 consecutive days with their breakfast meal, while participants in PG consumed a nitrate-free placebo from the same manufacturer (0.065-mmol NO3^-^/70-mL Beet It Sport; James White Drinks, Ipswich, United Kingdom) for the same amount of time. Placebo drinks had the exact same packaging, color, smell and taste as its nitrate-rich counterpart, so neither the athletes or the researchers could know who were consuming what. Participants were instructed to not use mouth rinse during the supplementation period.

### Physiological parameters

#### Incremental running test

The maximal graded test was performed with a computerized treadmill (H/P/COSMOS 3PW 4.0, H/P/Cosmos Sports & Medical, Nussdorf-Traunstein, Germany) to determine each subject’s maximal VO2, and to measure running economy at different speeds. Expired gases were measured breath-by-breath with the Jaeger Oxycon Pro gas analyser (Erich Jaeger, Viasys Healthcare, Germany). Heart response was continuously monitored with a 12-lead ECG. Participants started with 3 minutes warm-up at 10 km/h. Then the protocol continued with 3 steady state steps of 3 min each, at 15, 17.1 and 20 km/h. Subsequently the speed was increased to 0.2 km/h every 12 seconds until exhaustion. A slope of 1% was set throughout the test [24]. All the tests fulfilled at least 2 of the following criteria [2]: A respiratory exchange ratio (RER) >1.10, a plateau in VO2 (corresponding to a variation of, 100 ml*min^-1^) despite an increase in exercise intensity and a peak HR>220-age. This test was conducted at baseline and repeated 24-h after the 15th day of supplementation, so there were at least 24h after the last beetroot juice ingestion.

#### O2 saturation measurement

Throughout all the graded tests O_2_ saturation (SmO_2_, in %) was registered using near-infrared spectroscopy by placing a wearable device on the belly of the vastus laterallis (Moxy Monitor, Minnesota, USA). In this study, a wearable device was used to measure the muscle oxygenation in the vastus lateralis of dominant leg (along the vertical axis of the thigh, approximately 10–12 cm above the knee joint [25]. The device was wrapped by an elastic tight without occluding the blood flow. Black bandages covered the device to eliminate background light [26].

### Biomechanical parameters

To measure leg stiffness the validated Runmatic app v2.8 installed on an iPhone 6 with iOS 10.3.3 was used following the procedures described elsewhere [27]. The Runmatic app was used to record leg stiffness during the first minute of each steady state on the incremental test (i.e., at 15, 17.1 and 20 km/h), and mean scores were registered.

### Psychological parameters

At the completion of each steady state step of the incremental running test (i.e., during the last 10 seconds of the 3-min run at 15, 17.1 and 20 km/h) participants were asked to rate their RPE in a 1–10 scale. Before starting the incremental test, participants were given instruction to point to a number on a sheet held by a researcher corresponding to their RPE at each step during the trial. Participants were told that 1 means no exertion at all, and 10 means maximal exertion.

### Statistical analyses

Data is presented as mean with standard deviations. The non-parametric Mann-Whitney’s U statistical test was used to compare the pre-post differences between the experimental and placebo groups. Also, the standardized mean differences (SMD) with the corresponding 90% confidence interval (CI) were calculated using a magnitude-based inference approach [28]. The criteria for interpreting the magnitude of the SMD were: trivial (<0.2), small (0.2–0.6), moderate (0.6–1.0) and large (>1.0). Probabilities were also determined to establish whether true differences were lower, similar or higher than the smallest worthwhile change (0.2 x between–participant SD). Quantitative chances of better or worse effects were assessed qualitatively as follows: <1%, almost certainly not; 1–5%, very unlikely; 5–25%, unlikely; 25–75%, possible; 75–95%, likely; 95–99%, very likely; and >99%, almost certain. If the changes of better or worse were both >5%, the true difference was assessed as unclear. Statistical analyses were performed using a custom spreadsheet [28] and SPSS 24 for Mac. The level of asymptotic significance was set as p< 0.05.

## Results

**Fig 2:**
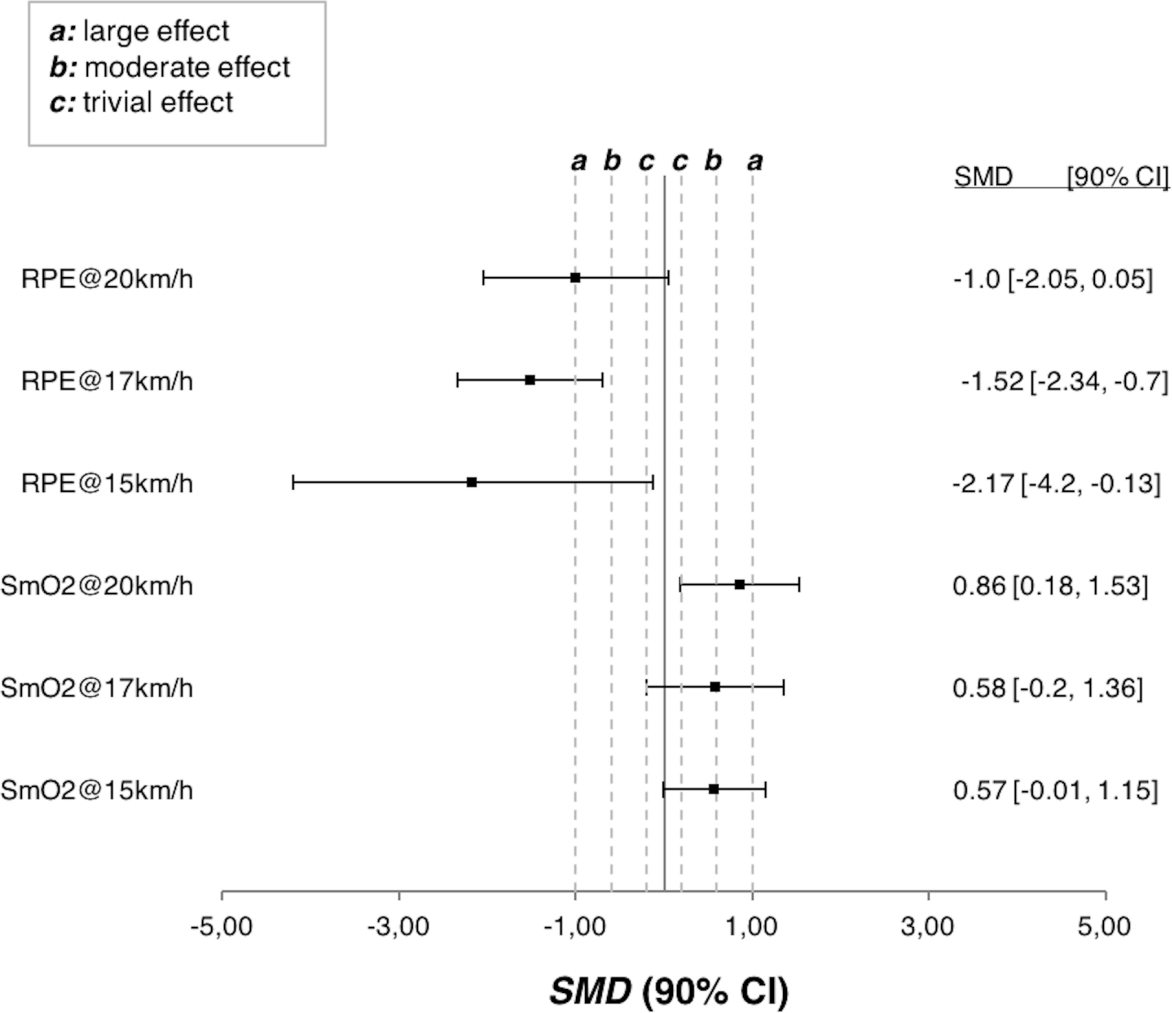
Forest plot with standardized mean differences (SMD) and 90% confidence interval (CI) for the rate of perceived exertion (RPE) and oxygen saturation (SmO_2_) at different running paces. Lower scores (i.e., to the left in the X-axis) means lower scores in the experimental group.

Participants in EG showed unclear improvements in heart rate (SMD = 0.03, 90%CI = -0.36, 0.42) and running economy expressed in VO2 (SMD = 0.36, 90%CI = -0.5, 1.21) in comparison with PG. Also, changes in the VO_2_Max did not substantially differ in EG in comparison with PG after the supplementation period (SMD = 0.04, 90%CI = -0.87, 0.95). However, likely to very likely improvements in EG vs. PG were observed for the RPE (SMD = -2.17, 90%CI = -3.23, -1.1), SmO_2_ (SMD = 0.72, 90%CI = 0.03, 1.41) (in the three steady stages) and time to exhaustion (SMD = 1.18, 90%CI = -0.14, 2.5). Finally, no substantial improvements in leg stiffness (SMD = -0.14, 90%CI = -0.44, 0.17) were observed during any of the steady stages of the incremental test as revealed by the low to trivial SMD and the range of the 90%CI. See Fig 2, Table 2 and Table 3 for more details.

**Table 2:** Mean values and standard deviations for the experimental and placebo groups before (Pre) and after (Post) the supplementation period.

	Experimental group		Placebo group	
	Pre	Post	Pre	Post
*Biomechanical parameters*				
Leg stiffness (kN/m)				
*15 km/h*	7.9 ± 0.9	8.6 ± 1.4	8.4 ± 1.2	8.5 ± 1.4
*17*.*1 km/h*	8.5 ± 1.2	8.7 ± 1.2	7.9 ± 1.1	8.3 ± 1.3
*20 km/h*	8.9 ± 0.8	9.2 ± 1.3	7.5 ± 1.0	8.1 ± 0.8
*Physiological parameters*				
RE (ml*kg*min^-1^)				
*15 km/h*	50.8 ± 3.5	52.6 ± 2.8	52.3 ± 3.3	53.7 ± 3.8
*17*.*1 km/h*	58.3 ± 2.7	60.4 ± 2.3	60.1 ± 3.8	62.1 ± 3.3
*20 km/h*	67.3 ± 4.4	69.5 ± 2.9	70.5 ± 5.1	71.4 ± 4.8
RER				
*15 km/h*	0.87 ± 0.02	0.83 ± 0.02	0.91 ± 0.02	0.87 ± 0.01
*17*.*1 km/h*	0.93 ± 0.02	0.90 ± 0.03	0.96 ± 0.02	0.94 ± 0.02
*20 km/h*	1.03 ± 0.01	0.97 ± 0.04	1.07 ± 0.03	1.03 ± 0.03
HR (bpm)				
*15 km/h*	147.2 ± 13.7	146.9 ± 11.2	157.3 ± 10.0	151.4 ± 12.4
*17*.*1 km/h*	160.6 ± 12.2	157.3 ± 9.2	168.1 ± 8.1	165.9 ± 7.1
*20 km/h*	164.2 ± 19.1	168.4 ± 11.4	164.6 ± 21.1	177.1 ± 4.6
SmO_2_ (%)				
*15 km/h*	41.7 ± 7.1	47.7 ± 3.9	46.6 ± 15.5	43.7 ± 5.9
*17*.*1 km/h*	34.4 ± 3.5	41.0 ± 2.0	38.2 ± 16.4	37.8 ± 5.2
*20 km/h*	24.4 ± 2.8	31.0 ± 6.9	26.8 ± 12.1	27.7 ± 4.8
TEx (s)	1173.0 ± 87.1	1269.0 ± 53.6	1251.0 ± 52.6	1230 ± 73.5
VO_2_Max (ml*kg*min^-1^)	69.1 ± 5.3	70.1 ± 7.0	72.3 ± 6.8	74.9 ± 6.1
*Psychological parameters*				
RPE				
*15 km/h*	2.4 ± 0.5	2.3 ± 0.5	2.4 ± 0.5	3.5 ± 0.8
*17*.*1 km/h*	3.8 ± 0.8	4.0 ± 0.8	4.4 ± 0.5	5.6 ± 0.8
*20 km/h*	6.0 ± 0.7	6.0 ± 1.0	7.2 ± 0.4	7.7 ± 1.0

Abbreviations: RE = running economy; VO2Max = maximal oxygen consumption; RER = respiratory exchange ratio; TEx = time to exhaustion; HR = heart rate; SmO2 = saturation of muscle O2; RPE = rate of perceived exertion

**Table 3:** Pre-post differences on the studied variables in the placebo vs experimental conditions.

	SMD (90%CI)	Chances of being beneficial/trivial/harmful	Qualitative inference	% of change (experimental vs placebo)	P
*Biomechanical parameters*					
Leg stiffness	-0.14 (-0.44, 0.17)	4/61/36	Possibly	2.25 vs 4.64	0.310
*Physiological parameters*					
RE	0.36 (-0.5, 1.21)	63/24/13	Unclear	7.07 vs 4.5	0.589
VO_2_Max	0.04 (-0.87, 0.95)	37/31/32	Unclear	4.41 vs. 3.66	1.000
Av_RER	-0.2 (-1.15, 0.74)	50/27/22	Unclear	-4.7 vs. -3.6	1.000
TEx (s)	1.18 (-0.14, 2.5)	90/6/4	Likely	8.18 vs 1.6	0.310
Av_HR	0.03 (-0.36, 0.42)	22/64/15	Unclear	-0.26 vs. 1.14	0.792
SmO_2_	0.72 (0.03, 1.41)	91/7/2	Likely	17.8 vs. -2.64	0.167
*Psychological parameters*					
RPE	-2.17 (-3.23, -1.1)	100/0/0	Very likely	-9.0 vs 20.4	0.016

Standardized mean differences (SMD) with 90% Confidence Intervals (CI) express the magnitude of the difference on the pre-post changes between the experimental and placebo groups. Positive values reflect higher increments on the placebo group after the supplementation period, while negative scores reflect lower increments on the experimental group after the supplementation period. P is the asymptotic significance of the Mann-Whiteny’s U test.

Abbreviations: RE = running economy; VO2Max = maximal oxygen consumption; Av_RER = average respiratory exchange ratio (whole test); TEx = time to exhaustion; Av_HR = average heart rate (whole test); SmO2 = saturation of muscle O2; RPE = rate of perceived exertion

## Discussion

The results of our study showed low to trivial improvements in different physiological measures such as running economy at different paces after 15-days of nitrate-rich beetroot juice supplementation in elite distance runners. These results are in line with other experiments conducted with elite distance athletes, in which submaximal exercise economy was not altered after the supplementation period either [20,29]. The supplementation period in our study was about twice larger than other experiments with elite runners; however, more days of ingestion of nitrate-rich beetroot juice supplementation doesn’t seem to boost the adaptations in submaximal exercise economy that this supplement has extensively showed to produce in less trained populations [17,18].

A novel contribution from our study was the analysis of the potential benefits of beetroot juice ingestion on different biomechanical variables that has been linked to running performance in the literature, such as leg stiffness [23,30]. For example, leg stiffness has showed to be negatively associated with running economy, since, in theory, a stiffer lower limb would store a higher amount of elastic energy that would assist in the force production during running and, therefore, it would reduce the cost of the exercise [9,23]. Results in our study showed trivial effects of beetroot juice supplementation in leg stiffness, similar to submaximal running economy.

Contrary to the results discussed above, likely to very likely large improvements were observed both in the rate of perceived exertion and the time to exhaustion in the experimental group in comparison with the placebo group after the supplementation period, where athletes who consumed nitrate-rich beetroot juice endured more time before voluntary stopping the incremental test on the treadmill. One potential explanation for this observation is the moderate increase in the SmO_2_ of the vastus lateralis of the athletes from the experimental group in comparison with the control group after the supplementation period. Participants who consumed nitrate-rich beetroot supplementation had larger percent of oxygen saturation in their muscles than their counterparts during exercise. That could have limited the accumulation of fatigue-related metabolites and reduce the depletion of PCr, which, in the end, could increase the time to exhaustion. NIRS is used for providing information about performance and oxygen muscle function and to evaluate response before, during and after exercise. In our study, the initial-during-end-exercise SmO_2_ was greater in experimental group. That finding is related with recovery capacity and improvements in performance status delaying the fatigue [31]. Then, greater availability of O_2_ could have impacted RPE and, finally it could have led to the observed increase in the time to exhaustion in the experimental group. For example, several studies have observed that increased oxygen availability via hyperoxia enhances exercise performance [32]. Nevertheless, further investigations are needed to better understand the mechanisms by which beetroot juice supplementation could reduce RPE and increase time to exhaustion in an incremental test on a treadmill in elite distance runners.

Summarizing, results in our study are in line with other investigations that observed no significant increases in running economy after a supplementation period with nitrate-rich beetroot juice in elite athletes [20,29]; however, the large increase in the time to exhaustion, the reduction in RPE and the increase in the oxygen saturation of the vastus lateralis during exercise, observed after 15 consecutive days of nitrate-rich beetroot supplementation provide novel information about the effects of this nutritional aid. Further investigations are required to confirm the findings of the present study, as well as to better understand the mechanisms behind these potential benefits for elite distance runners.

### Practical application and conclusions

Fifteen consecutive days of nitrate-rich beetroot juice supplementation didn’t increase running economy, locomotion mechanics or muscular power in elite distance runners. However, time to exhaustion in an incremental test on a treadmill, as well as rate of perceived exertion at different running paces were meaningfully higher and lower in the experimental group in comparison with the placebo group, respectively. These results might be explained in part by a higher muscle oxygen saturation observed in the vastus lateralis of the runners from the experimental group in comparison with the control group. Anyhow, one of the most performance-related variables in distance running (i.e., time to exhaustion) showed large improvements after 15-days of nitrate-rich beetroot juice supplementation. These results could have potential practical application for elite distance runners seeking nutritional strategies to improve their running performance.

## Supporting information

S1 File CONSORT 2010 checklist of information to include in a randomized controlled trial. (DOC)

S2 File Study protocol as approved by the ethics committee. (PDF)
