# Supplementary material for: The effects of beetroot juice supplementation on exercise economy, rating of perceived exertion and running mechanics in elite distance runners: A double-blinded, randomized study
Source: PLoS One. 2018 Jul 11;13(7):e0200517. doi: 10.1371/journal.pone.0200517 (PMC6040767; doi:10.1371/journal.pone.0200517)
Supplement: S2 File — (PDF) [file pone.0200517.s002.pdf]

UNIVERSIDAD CAMILO JOSÉ CELA

FORMULARIO DE SOLICITUD DE EVALUACIÓN POR EL COMITÉ ETICO DE INVESTIGACIÓN (CEI-UCJC)

Datos del Investigador Principal

Nombre Carlos Balsalobre Fernández

Facultad/Escuela Facultad de Formación de Profesorado y Educación, Universidad Autónoma de Madrid

Departamento Educación Física, Deporte y Motricidad Humana

Datos contacto carlos.balsalobre@icloud.com

Datos del Estudio de Investigación

Título del Proyecto The effects of nitrate supplementation via beetroot juice on the running economy, neuromuscular performance and running mechanics in elite middle and long distance runners / Los efectos de la suplementación de nitratos a través del zumo de remolacha en la economía de carrera, el rendimiento neuromuscular y la mecánica de carrera en corredores de media y larga distancia de élite (**ACRONIMO: BEET-RUN**)

La investigación incluye:

	Seleccionar	Apartado a completar
Solo seres humanos	X	Apartado A
Muestras biológicas		Apartado B
Organismos modificados genéticamente		Apartado C
Animales de experimentación		Apartado D

Documentación que se adjunta:

X	Formulario solicitud de evaluación
X	Apartados A - ~~B - C - D~~ (tachar los que no procedan)
X	COPIA DEL PROYECTO
X	Hoja de información a los participantes
X	Consentimiento informado a emplear
X	Compromiso escrito del Investigador Responsable

A completar por CEI-UCJC

Código de Proyecto:　　　　　　　　Fecha de Presentación/Versión:

Apartado A. Investigación en seres humanos sin toma de muestras biológicas

¿Qué grupos de participantes se han establecido? (indicar todos: enfermos, controles sanos menores, discapacitados…)

	**Describir**
Adultos sanos	X
Adultos enfermos	
Niños sanos	
Niños enfermos	
Discapacitados	
Mujeres embarazadas	
Mujeres lactantes	
Población en riesgo de exclusión social	
Otras poblaciones incapaces de expresar su consentimiento	
Grupos étnicos	

¿Por qué se han seleccionado dichos grupos?

Porque la investigación tiene como objetivo analizar un determinado tipo de suplementación nutricional (mediante zumos de remolacha) en el rendimiento en un grupo de corredores de élite.

¿Qué método de disociación de datos se va a utilizar? (según Ley 14/2007 de investigación biomédica)

	**Seleccionar el que proceda**
**Codificación (disociación reversible)**	X
**Anonimización (disociación irreversible)**	

Describir brevemente el procedimiento empleado para llevar a cabo dicha disociación

Los deportistas se numerarán de menor a mayor por orden alfabético. Posteriormente, cada variable que se mida tendrá un acrónimo, y en función de si dicha variable se ha medido en el momento pre o post intervención, se utilizarán las siglas PRE o POST. Por ejemplo, para identificar el valor de consumo de oxígeno pre-intervención del deportista ordenado en la lista en la posición 11, se utilizará el término “VO.11.Pre”

¿Se van a aplicar métodos invasivos para la toma de datos? En caso afirmativo, describa brevemente como se va a llevar a cabo dicho procedimiento estableciendo claramente las medidas relacionadas con la evitación del daño y la cualificación del personal que va a llevarlo a cabo.

NO PROCEDE. No se usarán métodos invasivos

En caso de producirse daño, ¿qué procedimiento paliativo/curativo se prevé realizar? ¿se cuenta con algún tipo de aseguramiento/compensación del daño? ¿Cuál? Si es que no explicar por qué no se ha establecido.

NO PROCEDE. No se usará métodos invasivos

¿Se ofrecen incentivos o compensaciones a los sujetos por su participación en los experimentos? Indique su naturaleza y cuantía.

NO

APPLICATION FORM FOR THE ETHICS COMMITEE APPROVAL (CEI-UCJC)

PRINCIPAL INVESTIGATOR

NAME Carlos Balsalobre Fernández

FACULTY Facultad de Formación de Profesorado y Educación, Universidad Autónoma de Madrid

Departament Educación Física, Deporte y Motricidad Humana

CONTACT INFORMATION carlos.balsalobre@icloud.com

INFORMATION OF THE TRIAL

TITLE The effects of nitrate supplementation via beetroot juice on the running economy, neuromuscular performance and running mechanics in elite middle and long distance runners / Los efectos de la suplementación de nitratos a través del zumo de remolacha en la economía de carrera, el rendimiento neuromuscular y la mecánica de carrera en corredores de media y larga distancia de élite (**ACRONIMO: BEET-RUN**)

RESEARCH INCLUDES:

	SELECT	SECTION TO COMPLETE
HUMANS	X	SECTION A
BIOLOGICAL SAMPLES		SECTION B
GENE MODIFICATIONS		SECTION C
EXPERIMENTS WITH ANIMALS		SECTION D

ATTACHED DOCUMENTATION:

X	APPLICATION FORM
X	SECTIO A - ~~B – C – D~~ (REMOVE WHAT DON'T PROCEED)
X	COPY OF THE APPLICATION FORM
X	INFORMATION FOR PARTICIPANTS ABOUT THE STUDY
X	INFORMED CONSENT
X	DECLARATION OF THE PRINCIPAL INVESTIGATOR

A completar por CEI-UCJC

Código de Proyecto: Fecha de Presentación/Versión:

SECTION A. INVESTIGATIONS WITH HUMAN BEINGS WITHOUT COLLECTING BIOLOGICAL SAMPLES

WHAT TYPE OF PARTICIPANTS ARE USED?

	SELECT
HEALTHY ADULTS	X
UNHEALTHY ADULTS	
HEALTHY KIDS	
UNHEALTHY KIDS	
DISABLE PEOPLE	
PREGNANT WOMA	
BREASTFEEDING WOMEN	
POPULATION AT RISK OF SOCIAL EXCLUSION	
OTHER POPULATIONS	
ETHNICAL SUBGROUPS	

WHY DID YOU SELECT THESE GROUPS?

Because the trial aims to analyze certain supplementation (beetroot juice) in the performance of elite runners

WHAT METHODS OF DISSOSIATION WILL YOU USE?

	SELECT
**CODIFICATION (reversible)**	X
**ANONIMATION (irreversible)**	

DESCRIBE THE PROTOCOL TO CONDUCT THAT DISSOSIATION

Athletes will be labeled with increasing numbers in an alphabetic order. Then, each variable will have an acronym, with PRE or POST depending of the moment at which it was measured. For example, to identify the pre-intervention VO2max of the athlete number 11, the term VO2Max.11.PRE will be used.

WILL YOU USE INVASIVE METHODS TO COLLECT THE DATA?

NO

IN THE EVENT OF PRODUCING DAMAGE, WHAT TREATMENT WILL YOU USE?

N/A. No invasive methods will be used

WILL YOU OFFER ANY REWARD TO THE SUBJECTS FOR THEIR PARTICIPATION IN THE EXPERIMENT?

NO
